# Transcription of IVIAT and Virulence Genes in *Photobacterium damselae* subsp. *piscicida* Infecting *Solea senegalensis*

**DOI:** 10.3390/microorganisms6030067

**Published:** 2018-07-12

**Authors:** José Alberto Núñez-Díaz, Milena Fumanal, Ana do Vale, Catalina Fernández-Díaz, Miguel Ángel Moriñigo, María Carmen Balebona

**Affiliations:** 1 Departamento de Microbiología, Universidad de Málaga, Andalucia Tech, Campus de Teatinos s/n, 29071 Málaga, Spain; mfumanal@uma.es (M.F.); morinigo@uma.es (M.Á.M.); 2 Fish Immunology and Vaccinology Group, IBMC-Instituto de Biologia Molecular e Celular, Universidade do Porto, 4200-135 Porto, Portugal; avale@ibmc.up.pt; 3 i3S-Instituto de Investigação e Inovação em Saúde, Universidade do Porto, 4200-135 Porto, Portugal; 4 IFAPA Centro El Toruño, Camino Tiro Pichón s/n, 11500 El Puerto de Santa María (Cádiz), Spain; catalina.fernandez.diaz@juntadeandalucia.es

**Keywords:** *Photobacterium damselae* subsp. *piscicida*, virulence, gene expression, in vivo, *Solea senegalensis*

## Abstract

*Photobacterium damselae* subsp. *piscicida* (*Phdp*) is responsible for disease outbreaks in marine aquaculture worldwide. *Solea senegalensis*, a valuable fish species for aquaculture in the south of Europe, is frequently affected by this pathogen. It is well established that bacteria respond to environmental signals and, in the case of pathogens, this ability may determine the outcome of their interaction with the host. Determination of gene expression under in vivo conditions constitutes a valuable tool in the assessment of microbial pathogenesis. Considering that different hosts may represent different environments for the pathogen, expression of *Phdp* virulence and in vivo induced antigen (IVIAT) genes during *S. senegalensis* infection has been determined in the present work. Increased transcription of genes encoding proteins involved in iron acquisition (Irp1, Irp2, HutB and HutD), oxidative stress defence (AhpC and Sod), adhesion (PDP_0080), toxins (AIP56) and metabolism (Impdh, Shmt and AlaRS) were detected in *Phdp* infecting *S. senegalensis* head kidney or liver. The highest increases corresponded to genes involved in survival under iron limiting conditions and oxidative stress, indicating their essential role during infection of sole. Results obtained give insight into *Phdp* virulence strategies and contribute to the identification of promising targets for the control of photobacteriosis.

## 1. Introduction

*Photobacterium damselae* subsp. *piscicida* (*Phdp*) is the causative agent of photobacteriosis. This pathogen has been reported to affect many fish species in marine worldwide aquaculture, especially in Mediterranean countries and Japan [1,2,3]. Virulence factors of this pathogen include a metalloprotease A-B exotoxin (AIP56) abundantly secreted by virulent strains [4,5,6].

AIP56 toxin induces apoptosis in fish macrophages and neutrophils, reducing phagocytic defence, favouring pathogen dissemination and promoting the release of phagocyte content causing tissue damage [5,7,8]. Apart from AIP56, another abundant protein detected in the extracellular products of *Phdp* is a 55 kDa protein (P55) identified as a NlpC/P60 family protein (Nuno MS dos Santos, personal communication). Although uncharacterized in *Phdp*, this family includes cell-wall related cysteine peptidases with homology to several Gram-negative bacterial proteins, many of them produced by pathogenic species.

Adhesion and invasion abilities are essential in the initial stages of several bacterial infections. *Phdp* has been reported to be weakly or moderately adhesive and invasive in some fish cell lines [9,10] and macrophages [11] and highly adhesive to intestinal cells [12]. A lipoprotein (PDP_0080) involved in the adherence of the bacterium to epithelial cells was identified [13] and vaccination of *Dicentrarchus labrax* with recombinant PDP_0080 lipoprotein resulted in increased survival when fish were challenged with *Phdp*. Nevertheless, information on the in vivo expression of virulence factors contributing to *Phdp* invasion of fish cells is still scarce.

The amount of free iron in infected hosts is extremely limited and, pathogens need to overcome this pitfall for the progress of the infection. *Phdp* is able to acquire iron from hemin and hemoglobin [14,15]. An heme uptake system encoded in nine genes arranged in two operons, *hutWXZ* and *tonBexbBDhutBCD*, allows *Phdp* to secrete proteins to extract heme from the heme-containing protein complex and deliver it to an outer membrane receptor [16]. Then, heme is transported into the periplasm by the TonB system, crossing finally the cytoplasmic membrane by an ATP-binding cassette system [17]. In this arrangement, *tonBexbBD* genes encode the components of the Ton system and *hutBCD* genes the periplasmic hemin binding protein, the inner membrane permease and the ABC transporter ATPase [16]. Furthermore, the ability to scavenge iron from the host by using high-affinity iron-binding siderophores has also been reported in *Phdp* [14,18]. A phenolate-like siderophore called piscibactin and encoded in a gene cluster resembling the *Yersinia* high pathogenicity island has been identified [19]. This siderophore is synthesized by means of a mechanism with participation of non-ribosomal peptide synthetases including one encoded in the *irp1* gene [20]. Recently, Núñez-Díaz et al. [21] detected induction of *Phdp irp1* expression during *S. senegalensis* infection by using in vivo induced antigen technology (IVIAT).

It is well established that bacteria respond to many different extracellular signals in the environment [22]. In the case of pathogens, an in vivo environment is sensed by invading bacteria that adapt by inducing or repressing specific genes allowing the pathogen to survive in the host and the progression of the infection [23]. In this way, bacterial cells have elicited a response to oxidative stress in order to diminish the damaging effects of reactive oxygen species (ROS) and reactive nitrogen species (RNS) produced by the host [24,25].

Several immunogenic proteins expressed by *P. damselae* subsp. *piscicida* during *S. senegalensis* infection have been identified using IVIAT [21]. These genes encode proteins such as inosine-5’-monophosphate dehydrogenase (Impdh), serine hydroxymethyl transferase (Shmt) and alanyl-tRNA synthethase (AlaRS), involved in aminoacid biogenesis and metabolism, the transfer of amino groups, and the uptake of carbohydrates from the extracellular environment. These three genes were not modulated during *Phdp* growth under iron-limiting or oxidative stress conditions. However, co-incubation of the pathogen with *S. senegalensis* kidney cells resulted in increased transcription, pointing to the in vivo induced character of these genes [21]. In addition, genes encoding the proteins alkyl hydroperoxide reductase (AhpC) and superoxide dismutase (Sod), both involved in the antioxidant activity, were identified. In this case, authors observed increased transcription in *Phdp* cells in contact with peroxynitrite, superoxide anions and *S. senegalensis* head kidney cells [21].

The development of control and prophylactic strategies requires the identification of pathogen components expressed during infection as well as the mechanisms involved in their regulation. In the present work, the transcription of virulence related genes and genes encoding immunogenic proteins expressed in vivo (IVIAT) during *S. senegalensis* infection has been studied. The influence of iron and oxidative stress on *Phdp* gene transcription has also been addressed.

## 2. Materials and Methods

### 2.1. Bacterial Strain

*Photobacterium damselae* subsp. *piscicida* (*Phdp*) (strain Lg41/01) was isolated from diseased cultured *S. senegalensis* [25] and cultured in tryptic soy broth (Oxoid Ltd., Basingstoke, UK) supplemented with 1.5% NaCl (TSBs) at 22 °C for 24 h. *Phdp* strain was stored at −80 °C in media supplemented with 15% glycerol.

### 2.2. Growth of Phdp under Iron Limiting Conditions and Oxidative Stress

The expression of virulence genes (*aip56*, *pdp-0080*, *hutB*, *hutD* and *p55*) in *Phdp* under in vitro culture and during in vivo infection was assessed by quantitative reverse transcription polymerase chain reaction (RT-qPCR). For in vitro culture, *Phdp* Lg41/01 was grown in TSBs at 22 °C and cells collected at mid-exponential (OD_600nm_ = 0.8) and stationary phase (OD_600nm_ = 1.4). Effect of iron availability on gene expression was determined in *Phdp* cultures grown in the presence of dipyridyl (100 µM) or FeCl_3_·6H_2_O (100 µM) at 22 °C until mid-exponential and stationary phase. Cultures in TSBs were used as controls. To study the effect of the oxidative stress on gene transcription, *Phdp* cells were grown in TSBs until mid-exponential phase and methyl viologen (0.2 mM), which generates superoxide radicals, was added. Cultures were incubated for further 6 h before centrifugation according to Díaz-Rosales et al. [26]. On the other hand, peroxynitrite (Calbiochem, Merck Millipore, Burlington, MA, USA) was added to mid-exponential phase cultures to achieve 1 mM final concentration and cells were recovered after 2 h. In both cases, cultures in TSBs were performed and used as controls. Survival of *Phdp* to oxidative stress treatments was confirmed previously. Triplicate cultures were carried out for each growth condition and cell pellets obtained after centrifugation (5000× *g*, 10 min, 4 °C) were frozen in liquid nitrogen and kept at −80 °C until analysis.

### 2.3. Solea senegalensis Infection with Phdp

A total of 60 *S. senegalensis* (54.2 ± 15.6 g mean body weight) specimens were challenged with *Phdp*. Fish were distributed in four 450-L tanks (15 specimens per tank) for experimental infection. Two duplicate groups were established: (1) specimens intraperitoneally injected with phosphate-buffered saline (PBS) and (2) fish intraperitoneally injected with *Phdp* suspended in PBS.

*Phdp* cells were grown in TSBs at 22 °C for 24 h and suspended in PBS (OD_600nm_ = 1). Fish were anaesthetized with clove oil (100 ppm) and injected with 0.1 mL of the bacterial suspension (dose 1 × 10^6^ CFU g^−1^). Then, the fish were returned to their respective tanks and mortality was recorded for 15 d. The control groups were inoculated with the same volume of sterile PBS. Mortality was considered due to the pathogen when *Phdp* was isolated from internal organs of dead fish. *Phdp* detection was determined in head kidney and liver by PCR according to Osorio et al. [27], and using tryptic soy broth (Oxoid Ltd., Basingstoke, UK) supplemented with 1.5% NaCl (TSAs) at 22 °C for 48 h.

According to previous studies, mortality was expected 96 h post-infection. For this reason, three fish were randomly sampled from one tank of each group (infected and control groups) at this time. Mortality in both infected and control fish was recorded in the other replicate tanks. Infected *S. senegalensis* were euthanized and the head kidney and liver sampled. All the samples were immediately submerged in TRIsure (Bioline, London, UK) and stored at −80 °C.

### 2.4. Bacteria Gene Expression Analysis

Total RNA from *Phdp* cells grown in different conditions was extracted with TRIsure according to the manufacturer's instructions. RNA quality was checked by running an aliquot on an agarose gel and quantity spectrophotometrically determined in Nanodrop ND-1000 (Thermo Fisher Scientific, Madrid, Spain) via A_260/280nm_ and A_260/230nm_ readings. DNase treatment (Thermo Scientific) was carried out to ensure complete removal of DNA. Reverse transcription was performed using First Strand cDNA Synthesis Kit (Thermo Fisher Scientific, Madrid, Spain) with 1 µg of total RNA. One microliter of each cDNA synthesis reaction was employed as the template in the RT-qPCR reactions to analyse gene transcription. Three biological and technical replicates were used for our experiments. Relative transcription of genes encoding AIP56, HutB, HutD, P55, the lipoprotein PDP_0080, Sod, AhpC, Impdh, Irp1, Irp2, Shmt and AlaRS was determined using qRT-PCR and *16S rRNA* was used as reference gene according to Núñez-Díaz et al. [21]. Specific primers for amplification of genes encoding AIP56, HutB, HutD, P55, and the lipoprotein PDP_0080 were designed in this study by using Primer 3 and AmplifX software according to known RT-qPCR restrictions (size, Tm difference between primers, % GC content and self-dimer or cross-dimer formation). In order to obtain accurate results, PCR efficiency was checked to ensure optimized and reproducible assays.

$$E=\left(10^{\left[-1/\mathrm{slope}\right]}-1\right)\times 100$$

RT-qPCR reactions were performed in a CFX96 Touch Real-Time PCR Detection System (Bio-Rad Laboratories, Hercules, CA, USA) with an initial denaturation cycle of 95 °C for 60 s, followed by 40 cycles of 95 °C for 30 s, 55 °C for 40 s and 72 °C for 60 s. Amplification was followed by a standard melting curve from 65 °C to 95 °C, in increments of 0.5 °C for 5 s at each step, to confirm that only one product was amplified and detected. Samples were run in parallel with *16S rRNA* reference gene. The change in gene expression in the different growth conditions was recorded as comparative Ct (2^−∆∆*C*t^) [28] normalized to the reference gene and relative to cells grown in TSBs. Primers used for the genes assayed in this work are summarized in Table 1.

### 2.5. Bacteria Gene Expression Analysis under In Vivo Conditions

To study the effect of the in vivo environment on *Phdp* gene transcription, liver and head kidney from three infected *S. senegalensis* specimens (dose 1 × 10^6^ CFU g^−1^) were individually isolated and RNA extracted using TriSure (Bioline) according to the manufacturer’s protocols. DNase treatment and reverse transcription was performed following the methodology previously detailed. Transcription under in vivo conditions was determined by RT-qPCR using *16S rRNA* for normalization and relative to *Phdp* cells grown in TSBs. Liver and head kidney from non-infected fish (control group) were isolated and processed as described above to check the absence of *Phdp* gene expression.

### 2.6. Statistical Analysis

Statistical analysis was performed using XLSTAT v2014.5.03 (Addinsoft, New York, NY, USA) for Microsoft Excel (Microsoft Corporation, Redmond, WA, USA). Results are shown as means ± standard errors of the mean (SEM). Normality and homogeneity of the data were previously assessed using Shapiro–Wilk and Levene tests, respectively. For non-normal data, a logarithmic transformation was performed. The statistical significance of differences in RT-qPCR values between control and treated groups was determined by one-way analysis of variance (ANOVA). Tukey’s test was used to analyse differences between the treatments. Significance was set for *p* < 0.05.

### 2.7. Ethical Statements

All studies involving fish were conducted in strict accordance with guidelines established by the European Union (2010/63/UE) and the Spanish legislation (RD216 1201/2005 and RD 53/2013) for the use of laboratory animals. All procedures were authorized by the Bioethics and Animal Welfare Committee of the Institute of Agricultural and Fisheries Research and Training (IFAPA), and given the registration number 17/11/2016/171 (17 November 2017) according to the national authorities for regulation of animal care and experimentation.

## 3. Results

### 3.1. In Vitro Transcription of Virulence Genes

**Figure 1:**
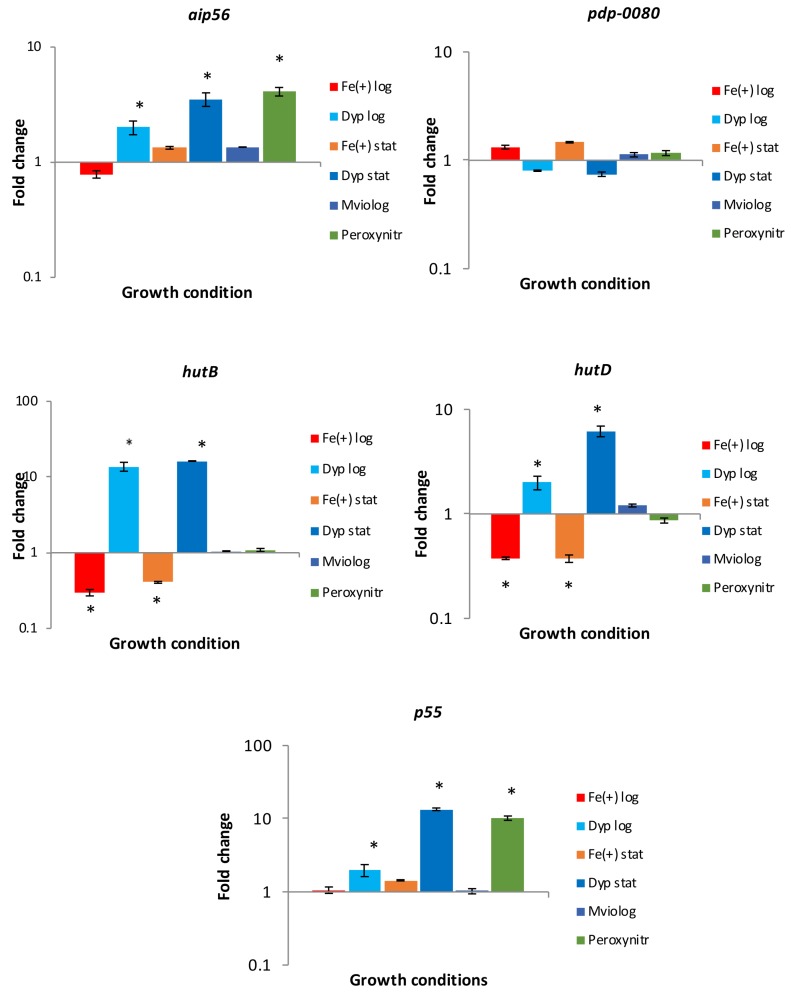
Relative transcription of *Phdp* genes *aip56*, *pdp-0080*, *hutB*, *hutD* and *p55* in *Phdp* cells grown under iron-replete or limiting conditions and exposed to superoxide and peroxynitrite radicals. Fe (+) log and Fe (+) stat: *Phdp* cells were grown in TSBs supplemented with FeCl_3_ (100 µM) until log or stationary phase, respectively. Dyp log and Dyp stat: *Phdp* cells were grown in TSBs containing 2,2′-dipyridyl (100 µM) until log or stationary phase, respectively. MViolog: *Phdp* cells were grown until log phase and then incubated for 6 h in the presence of methyl viologen (0.2 mM). Peroxynitr: *Phdp* cells were grown until log phase and then incubated for 2 h in the presence of peroxynitrite (1 mM). Quantitative polymerase chain reaction (RT-qPCR) data were normalized against *16S rRNA* gene and fold change values calculated at each sampling time relative to non-treated cells (grown in TSBs) based on the 2^−ΔΔ*C*t^ method. Values represent the mean ± standard error of the mean (SEM) of three independent experiments. Significant differences (*p* < 0.05) compared to non-treated cells have been indicated with an asterisk (*).

Expressions of selected genes by *Phdp* cells grown under iron–limiting and replete conditions were analysed with RT-qPCR. Results showed up-regulation of genes encoding the toxin AIP56, the protein P55 and the hemin binding and transport HutB and HutD proteins in cells grown under iron-limiting conditions until log or stationary phase (Figure 1). Increased relative transcription observed in iron limiting conditions was more noticeable in stationary phase cultures compared to log phase in the case of *aip56*, *p55* and *hutD* genes, whilst no difference related to the growth phase was observed in *hutB*. Furthermore, *hutB* and *hutD* genes were down-regulated in bacterial cells grown under high iron concentrations. On the contrary, no modulation by iron availability was observed in the gene encoding the lipoprotein PDP_0080 (Figure 1).

Reactive oxygen and nitrogen species can also be encountered by pathogens during host infection. Oxidative stress due to superoxide anions produced by methyl viologen did not modulate the transcription of assayed genes; however, *aip56* and *p55* genes were up-regulated by peroxynitrite, whilst no significant changes were observed in *hutB*, *hutD* and *pdp-0080* (Figure 1).

### 3.2. Experimental Infection

**Figure 2:**
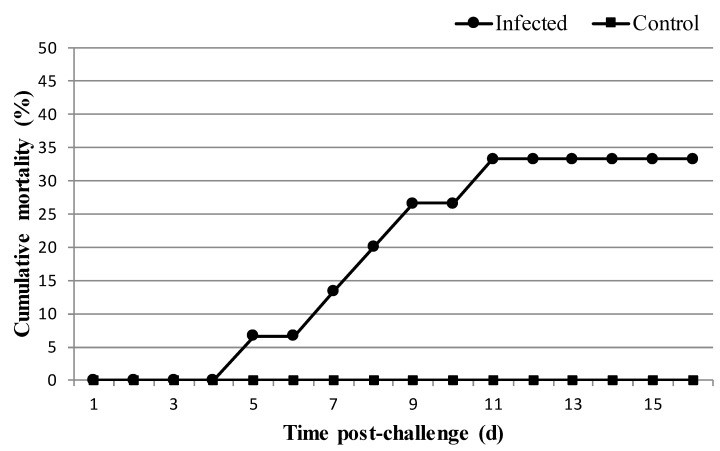
Cumulative mortality in infected and control *S. senegalensis* groups after challenge with *P. damselae* subsp. *piscicida* (*n* = 15 per group).

*Phdp* PCR detection and bacteriological analysis of fish were carried out before experimental infection with negative results. Mortality was observed only in the infected group and started 4 days after infection, with maximum (33% cumulative mortality) reaching 9 days post-infection (Figure 2). Afterwards, no mortality was recorded until the end of the experiment. Dead fish were analysed and the presence of *Phdp* was confirmed by PCR and bacteriological assays.

### 3.3. In Vivo Transcription of Phdp Virulence Genes

Samples from non-infected *S. senegalensis* specimens were negative for amplification of *Phdp* genes assayed in this study.

**Figure 3:**
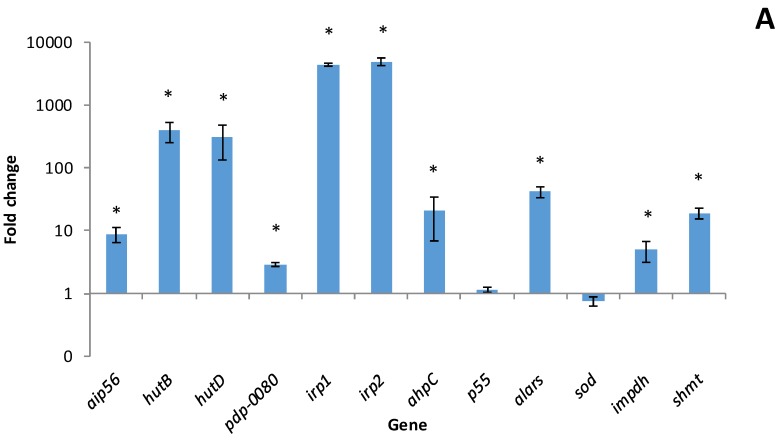

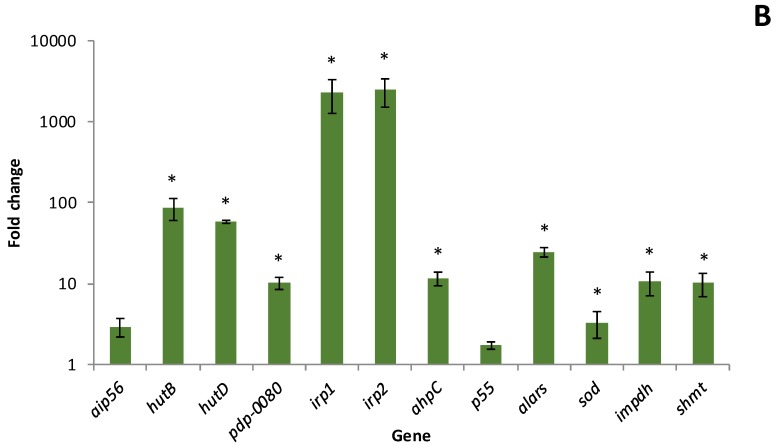
Relative transcription based on the 2^−ΔΔ*C*t^ method of *Phdp* genes in liver (**A**) and head kidney (**B**) of *S. senegalensis* specimens 96 h post-infection. Data were normalized against *16S rRNA* gene and *Phdp* cells grown in bacteriological media were used for fold change calculations. Values represent the mean ± SEM of three fish. Significant differences (*p* < 0.05) compared to in vitro grown bacterial cells have been indicated with an asterisk (*).

After 96h of infection with *Phdp*, up-regulation of *pdp-0080*, *hutB*, *hutD*, *irp1*, *irp2*, *ahpC*, *alars*, *impdh* and *shmt* genes was detected in *Phdp* cells in both *S. senegalensis* liver and head kidney compared to in vitro grown bacterial cells (Figure 3). Genes *irp1*, *irp2*, *hutB* and *hutD* that are known to be involved in iron acquisition from the host [16,20] showed the highest up-regulation rates. AIP56 encoding gene exhibited significant up-regulation only in the liver; on the contrary, the antioxidant Sod protein gene showed up-regulation in the head kidney. Finally, no significant differences were detected in the organs assayed for *p55* gene transcription compared to in vitro levels (Figure 3).

## 4. Discussion

Organisms react to environmental stimuli with different responses, including the modulation of gene expression, to improve their ability to proliferate in a specific environment. Bacteria invading a host sense an in vivo environment and adapt by inducing or repressing the expression of a set of genes. In the case of pathogens, understanding the influence of host environmental components regulating virulence gene transcription is essential in order to develop strategies to control pathogen infection [23].

Iron is essential for organisms, with critical functions in many cellular processes. However, this metal can also generate reactive oxygen species (ROS) via the Fenton reaction [29]. ROS are generated not only during the course of normal prokaryotic cellular homeostasis but also by host cells in response to bacterial infection [30]. For these reasons, bacteria must tightly control the uptake and storage of iron in a manner that restricts the build-up of endogenous ROS and must adapt to resist to the oxidative radicals generated during respiratory burst.

The present work showed that iron availability regulates transcription of *hutB* and *hutD* in *Phdp*. The expression of these genes was also up-regulated in the pathogen infecting the liver and head kidney of *S. senegalensis* compared to in vitro cultured *Phdp* cells. Although not compared to in vitro levels, expression of haem uptake genes has already been described in *Phdp* infecting *Psetta maxima* [18]. On the other hand, *irp1* and *irp2* genes are involved in the synthesis of the siderophore piscibactin [18]. The *irp1* gene encodes an in vivo induced protein detected in *S. senegalensis* infected with *Phdp* and growth of *Phdp* under iron-restricted conditions that resulted in the up-regulation of *irp1* and *irp2* genes [21]. In this way, increased transcription of genes encoding proteins such as hepcidin, ferritin and transferrin involved in the regulation of iron availability has been reported in the liver and kidney of *S. senegalensis* [31] and *D. labrax* [32] during *Phdp* infection. This response leads to low iron availability and depression of serum iron levels [33]. Increased transcription of *hutB, hutD, irp1* and *irp2* observed in bacteria infecting the liver and head kidney of Senegalese sole suggests that the pathogen is responding to the host environment by promoting mechanisms such as hemin utilization and siderophore synthesis to overcome iron starvation inside the host [14,17,34].

It has been previously shown that expression of *irp1* and *irp2* is up-regulated when *Phdp* is subjected to oxidative stress induced by superoxide and peroxynitrite [21]. *Phdp* Sod has been described as an iron co-factored protein and lower transcription and activity levels of Sod were detected in the pathogen growing under iron-limiting conditions [21,24]. Similar behaviour has been described in other pathogens such as *H. pylori*, where *sodB* transcription is directly regulated by a Fur protein [35]. Transcription levels of *Phdp sod* in the liver of *S. senegalensis* during infection were similar to those observed in bacteriological medium, under in vitro conditions, whereas up-regulation was observed in the bacterial cells infecting the head kidney. Differences in the environments present in these organs could explain these results; however, further studies are necessary to clarify this point. Results obtained in the present work indicate increased *sod* transcription during infection, with relative rates in the head kidney higher to those observed in bacteriological media. *Phdp* has iron-acquisition systems expressed during host infection [14,20]. They could provide the pathogen enough iron to survive the iron-limiting conditions encountered in the host but not be enough to induce *sod* transcription in the liver.

Conversely, iron-limiting conditions as well as high ROS and RNS induced higher *ahpC* transcription [21] and *ahpC* up-regulation was also observed during *S. senegalensis* infection. Increased *ahpC* transcription under iron-limiting conditions has been previously reported in *Corynebacterium diphtheria*, *Bacillus subtilis* and *Campylobacter jejuni* [36]. In this situation, the production of alkyl hydroperoxide reductase coupled to the activation of iron acquisition mechanisms, can contribute to pathogen survival, when transient increases of intracellular iron concentrations carries the risk of oxidative stress.

AIP56 is a key virulence factor in photobacteriosis because macrophage and neutrophil apoptosis triggered by AIP56 reduces host phagocytic cells involved in the restriction of *Phdp* multiplication [4,6,37,38]. Results obtained in the present work show increased transcription of AIP56 encoding gene during *Phdp* infection of *S. senegalensis*. Production of the toxin during infection in sea bass (*Dicentrarchus labrax*) has already been reported [5]. In the present work, increased *aip56* transcription has been observed in *S. senegalensis* liver during infection assays compared to in vitro conditions using bacteriological media, whilst no changes were detected in the head kidney. The fact that low iron availability and oxidative stress due to peroxynitrite also results in increased transcription, points to a regulation by these conditions normally encountered by the bacteria during host infection [33]. Differential conditions present in the liver and head kidney leading to an absence of *aip56* up-regulation in the head kidney need further research.

P55, one of the more abundant protein excreted by *Phdp* has been identified as a NlpC/P60 containing protein (Nuno MS dos Santos, personal communication) homologous to several Gram-negative bacterial proteins present in highly invasive pathogens such as *Salmonella enterica* [39] and demonstrated to be required for pathogenesis in *Mycobacterium tuberculosis* [40]. Proteins containing NlpC/P60 domains work as bacterial hydrolases and contribute to cell wall remodelling during zebrafish infection caused by *Mycobacterium marinum* [41,42]. In the present work, the regulation of the gene encoding P55 protein by iron and peroxynitrite was similar to the one observed for the AIP56 encoding gene. However, no changes in *p55* gene transcription were detected in *Phdp* colonizing *S. senegalensis* liver or head kidney 96 h post-infection. Time-course experiments to elucidate potential regulation of virulence along the infectious process need to be carried out. In the present work, samples were analysed 96 h post infection, just before the onset of mortality, but changes in *Phdp* virulence gene expression at early or later times could be expected, allowing the pathogen to adapt to host response. In this way, further studies on the role of P55 protein in virulence will indicate if the protein is regulated in the initial stages of the infection.

*Phdp* is considered as a facultative intracellular pathogen capable of entering and surviving in both fish phagocytic and non-phagocytic cells [9,10,43,44]. The lipoprotein PDP_0080 has been reported as involved in *Phdp* adhesion to fish cells [13]. Transcription of the gene encoding this protein was not regulated by iron levels or oxidative stress. However, up-regulation was observed during *Phdp* infecting *S. senegalensis* liver and head kidney. Bacterial lipoproteins play a wide range of functions in the interaction with the host, including adhesion, translocation as well as evasion of the immune system [45,46]. Results obtained indicate that although low iron availability or oxidative stress did not affect *PDP_0080* gene expression, other factors encountered by the bacteria in *S. senegalensis* were able to up-regulate gene transcription of this protein involved in *Phdp* adhesion.

Regarding genes encoding proteins involved in nucleotide biogenesis and metabolism (*impdh*) [47,48], amynoacylation of unfinished polypeptides (*alars*) [49] and incorporation of carbohydrates from the extracellular milieu for biomolecules biosynthesis (*shmt*) [50], in vivo up-regulation is in agreement with previous studies performed in a co-incubation assay of *Phdp* with head kidney cells of *S. senegalensis* [21]. In this context, increased in vivo transcription of these metabolism-related genes suggests that the proteins encoded may play a role in *Phdp* pathogenicity. Studies revealed that treatment of *Vibrio parahaemolyticus* with antimicrobial peptides leads to down-regulation of the *impdh* gene, causing a reduction in nucleotide metabolism, probably for energy preservation [48], and attenuation of virulence in *Streptococcus suis* type 2 mutants lacking *impdh* was observed [51]. Concerning the *shmt* gene, works in *Vibrio cholerae* [52] and *Salmonella typhimurium* [53] showed virulence reduction with this gene mutated. Additionally, bacterial species such as *Haemophilus influenzae* with mutations in the *alars* gene exhibited a reduction in survival [54], suggesting an important role for this gene in pathogenicity.

Briefly, the results reported here indicate that *Phdp* genes related to toxin production (*aip56*), iron acquisition (*irp1*, *irp2*, *hutB* and *hutD*), antioxidant activities (*sod* and *ahpC*), adhesion to cells (*pdp_0080*), and metabolism (*impdh*, *shmt* and *alars*) are up-regulated during *S. senegalensis* infection. This knowledge of the regulation of genes involved in virulence is essential for the development of therapeutic and preventive strategies for photobacteriosis, as proteins encoded by genes that were found to be up-regulated in vivo can now be considered as valuable targets for vaccine and treatment formulations.

## Figures and Tables

**Table 1:** List of primers used in the present study.

Gene	Code	Sequence (5’→3’)	Amplicon Size (bp)	Source
Apoptosis induced protein 56 kD	*aip56*	GGTCGAAGCGATACAAGAGC (F) CCGTTGAAATCATCATCGTG (R)	207	This study
Adhesion lipoprotein	*pdp-0080*	TGCAGGCCAACATCTAACAG (F) TTAGCTCAGCAGGGAATGGT (R)	158	This study
periplasmic hemin binding protein	*hutB*	ACGGAGCATCGTTCTCAACT (F) TGGCATTGTTTTGATGGTTG (R)	264	This study
ABC transporter ATPase	*hutD*	TGAACCCACATCTGCTCTTG (F) GCGGTTGGGGTTAGTACTTG (R)	201	This study
Protein 55 kD	*p55*	GGATTTGGCTACCTCGTTCA (F) CCCACGGAGCATTAAACATT (R)	249	This study
Alkyl hydroperoxide reductase	*ahpC*	ATGGTGGTATTGGCCCTGTT (F) CATTGAGCTGGGCACACTTC (R)	250	[21]
Inosine-5’-monophosphate dehydrogenase	*impdh*	TGCTGATGGTGGTATCCGTT (F) GACATCGCACCAAGAGAACC (R)	177	[21]
Superoxide dismutase	*sod*	AGACGCACTAGAACCACACA (F) GGGCTTAGACAGTGCCAGTA (R)	213	[21]
Non-ribosomal peptide synthetase involved in siderophore biosynthesis 1	*irp1*	GCTACAGAGGCCGCTATTTG (F) CTTCATCTTGCCAGTAGCCA (R)	202	[21]
Non-ribosomal peptide synthetase involved in siderophore biosynthesis 2	*irp2*	AGGCAGCATTTCAGCAGATT (F) CGTTGTTCTCGGTCGGTATT (R)	226	[21]
Serine hydroxymethyl transferase	*shmt*	CGGAACTTTATGCAGCCATT (F) CAATGGCAAGTTGTTCTGCT (R)	201	[21]
Alanyl-trna synthethase	*alars*	GTGTTAAGCATGGGCGATTT (F) CCTTGTTCACCACAGAAGCA (R)	232	[21]
16S ribosomal RNA	*16S rRNA*	AACTGGCAGGCTAGAGTCTT (F) CACAACCTCCAAGTAGACAT (R)	198	[21]
